# Utility of traditional and non-traditional lipid indicators in the diagnosis of nonalcoholic fatty liver disease in a Japanese population

**DOI:** 10.1186/s12944-022-01712-z

**Published:** 2022-10-07

**Authors:** Song Lu, Maobin Kuang, Jinjing Yue, Chong Hu, Guotai Sheng, Yang Zou

**Affiliations:** 1 Medical College of Nanchang University, 330006 Nanchang, Jiangxi Provincial China; 2 Jiangxi Cardiovascular Research Institute, Jiangxi Provincial People’s Hospital, 330006 Nanchang, Jiangxi Provincial China; 3 Department of Obstetrics and Gynaecology, The Third Affiliated Hospital of Zhengzhou University, 450052 Zhengzhou, Henan Provincial China; 4 Department of Gastroenterology, Jiangxi Provincial People’s Hospital, 330006 Nanchang, Jiangxi Provincial China

**Keywords:** Non-traditional lipid indicators, RC/HDL-C ratio, TG/HDL-C ratio, Nonalcoholic fatty liver disease

## Abstract

**Background:**

Traditional and non-traditional (TNNT) lipid indicators are known to be closely related to nonalcoholic fatty liver disease (NAFLD). This study’s objective was to compare the degree of associations and diagnostic values of TNNT lipid indicators with NAFLD.

**Methods:**

Participants were 14,251 Japanese adults who undergoing health checkups, and we measured and calculated 11 lipid indicators, including traditional lipid indicators such as high-density lipoprotein cholesterol (HDL-C), total cholesterol (TC), low-density lipoprotein cholesterol (LDL-C), and triglyceride (TG), as well as non-traditional lipid indicators such as TC/HDL-C ratio, LDL-C/HDL-C ratio, TG/HDL-C ratio, non-HDL-C, remnant cholesterol (RC), RC/HDL-C ratio and non-HDL-C/HDL-C ratio. The associations between these lipid indicators and NAFLD were assessed using multivariate logistic regression, and the performance of these lipid indicators in identifying NAFLD was analyzed by receiver operating characteristic (ROC) curves.

**Results:**

After rigorous adjustment for potential confounders, multivariate logistic regression showed that all TNNT lipid indicators were independently associated with NAFLD, among which the RC/HDL-C ratio and RC had the strongest association with NAFLD. ROC analysis showed that non-traditional lipid indicators were superior to traditional lipid indicators in identifying NAFLD, especially in young adults and females. It is worth mentioning that the RC/HDL-C ratio was the best lipid indicator for identifying NAFLD with an area under the curve (AUC) of 0.82 and an optimal cut-off value of 0.43; in addition, TG/HDL-C ratio also had a high recognition performance for NAFLD.

**Conclusion:**

Overall, in the Japanese population, non-traditional lipid indicators had a higher diagnostic value for NAFLD compared to traditional lipid indicators, and lipid indicators alone had a lower diagnostic value for NAFLD than the ratio of two lipid indicators, with RC/HDL-C and TG/HDL-C being the best lipid indicators for identifying NAFLD.

**Supplementary Information:**

The online version contains supplementary material available at 10.1186/s12944-022-01712-z.

## Background

NAFLD, a pathological syndrome, comprises a series of liver lesions like simple steatosis, non-alcoholic steatohepatitis, cirrhosis, and liver cancer [1]. The prevalence of NAFLD has increased rapidly, in recent years, in the context of the rapid expansion of the global obesity and diabetes population and currently exceeds 25% of the global adult population, with the overall prevalence of NAFLD expected to increase to 33.5% in adults worldwide by 2030 [2, 3]. NAFLD will impose an additional financial burden of about 35 billion euros on the four European countries (Germany, United Kingdom, France, and Italy), and bring about $103 billion of economic losses to the United States [4], which has become an increasingly serious global public health problem [2, 3]. In addition, the increased prevalence of NAFLD also significantly increases the risk of chronic kidney disease, cardiovascular disease, glucose metabolism disorders, and malignancies (colorectal cancer and liver cancer) [5–8]. Therefore, early identification and diagnosis of NAFLD and intervention of potential risk factors to reduce the NAFLD risk in the population may be an ideal preventive strategy.

Abnormal lipid metabolism is the main risk factor for NAFLD [9, 10]. Patients with NAFLD usually exhibit atherogenic dyslipidemia, including elevated levels of TG and LDL-C and reduced HDL-C concentrations in traditional lipid indicators [11, 12], and exhibit increased RC and non-HDL-C levels, in non-traditional lipid indicators, as well as elevated TC/HDL-C ratio, LDL-C/HDL-C ratio, TG/HDL-C ratio, non-HDL-C, RC, RC/HDL-C ratio and non-HDL-C/HDL-C ratio [13–15]. Numerous epidemiological studies have also pointed out that these lipid indicators are very useful biomarkers for the identification of NAFLD [13, 14, 16–25]. Based on this background, it would be of great clinical importance to further identify the best lipid indicators for identifying patients with NAFLD, which would provide a great convenience for rapid screening of NAFLD. However, there is no clear statement on which lipid indicators are the most valuable ones to be used to identify NAFLD. Therefore, in the present research, we reported the utility of all the above TNNT lipid indicators as a screening tool for identifying patients with NAFLD, based on data from the NAGALA (NAfld in Gifu Region, Japan, Longitudinal Analysis) cohort of 14,251 subjects.

## Methods

### Data sources and study population

**Fig. 1:**
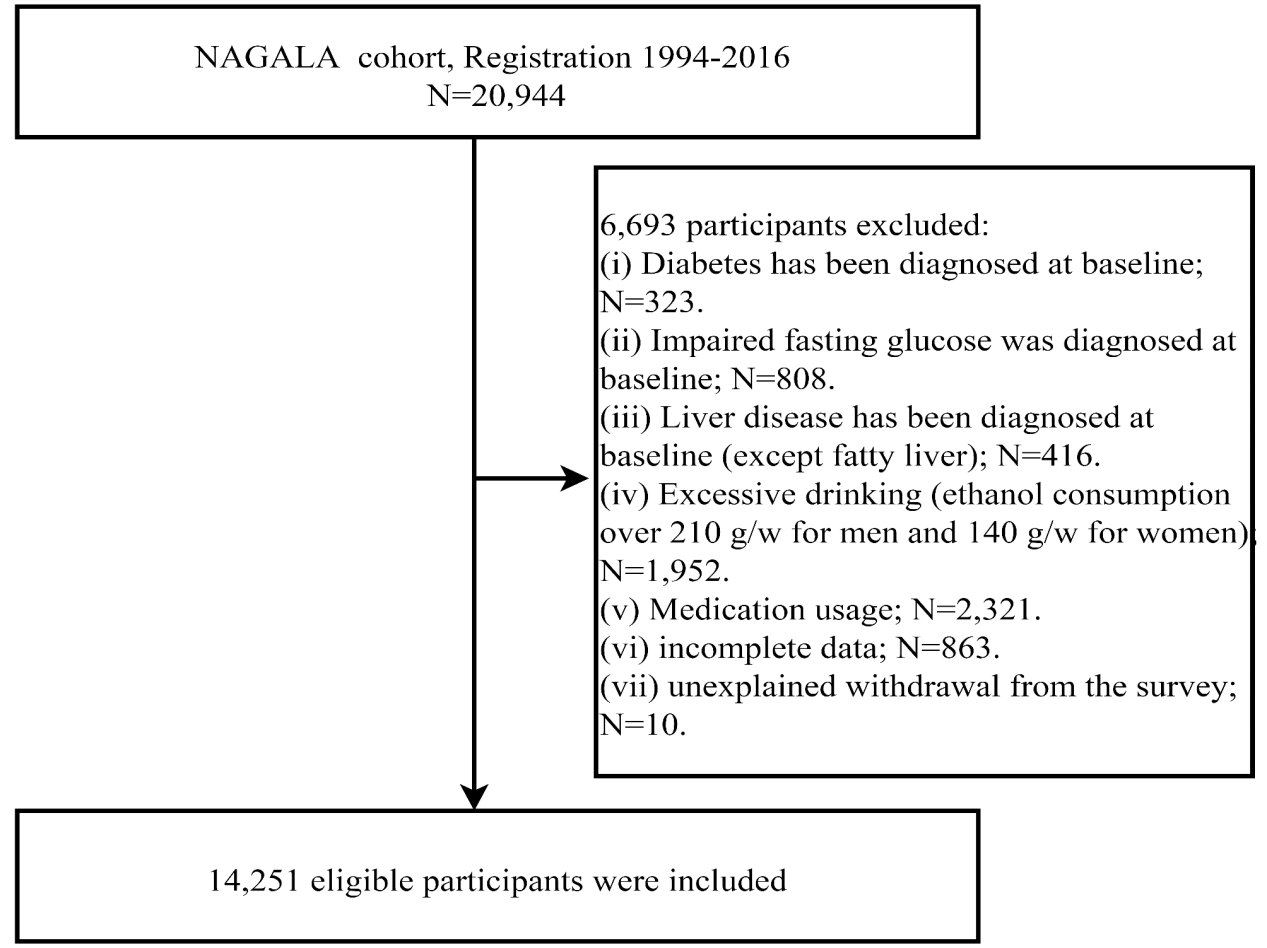
Study profile

In this study, we analyzed the NAGALA dataset to further evaluate the ability of TNNT lipid indicators to distinguish NAFLD. Details on the study design, participant registration, eligibility requirements, and data collection of the NAGALA cohort study population could be found in the original article (Okamura et al., 2019) [26]. Briefly, NAGALA is a longitudinal survey initiated by Murakami Memorial Hospital in 1994 to conduct population-based research on common chronic diseases and to promote public health. The NAGALA cohort data analyzed in the current research have been uploaded to the public database by Okamura et al. Based on the service terms of the Dryad database, we extracted the physical examination data of 20,944 adult subjects (ages: 18–79 years old) in the NAGALA dataset from 1994 to 2015 and performed a post-hoc analysis of these data according to the new research hypothesis. For this study, we excluded subjects with the following characteristics: (1) subjects with known liver disease (n = 416); (2) subjects with known diabetes or impaired fasting glucose (n = 1,131); (3) excessive drinkers: the total amount of alcohol consumed per week was ≥ 210 g for males and ≥ 140 g for females (n = 1,952) [27]; (4) subjects were taking medication at baseline (n = 2,321); (5) subjects with missing covariate data (n = 873). Finally, 14,251 subjects were included in this study (Fig. 1). In the previous study, the study protocol was authorized by the Murakami Memorial Hospital Institutional Review Board (IRB) and obtained the informed consent of the participants [26], and the current study, a post-hoc analysis of data from the NAGALA cohort, was approved by the IRB of Jiangxi Provincial People’s Hospital (IRB number:2021-066). Because the current data set has de-identified the identification information of the subjects, the requirement of informed consent has been abandoned.

### Data collection and measurement

As mentioned previously [26], information on subjects’ anthropometric indicators [waist circumference (WC), height, arterial blood pressure, weight, and body mass index (BMI)], lifestyle habits (smoking, drinking, and exercise habits) age, and sex were collected and recorded by trained health workers. According to the Asian BMI classification criteria (18.5/25), we classified the subjects’ BMI as low weight, normal weight, and overweight/obese [28]. According to the average amount and type of alcohol consumed by participants in the past month, we divided their drinking status into three groups: no/rarely drinking, little drinking, and moderate drinking. Similarly, according to the smoking history of the subjects, the smoking status was also divided into three groups: never, past and present. Moreover, exercise habits were defined as subjects participating in any form of physical activity at least once a week. Blood samples for the analysis of biochemical parameters were collected at least 8 h after fasting and analyzed for the determination of alanine aminotransferase (ALT), glycosylated hemoglobin A1c (HbA1c), aspartate aminotransferase (AST), HDL-C, TC, γ-glutamyl transferase (GGT), fasting plasma glucose (FPG), and TG using an automated analyzer according to standard methods.

**Fig. 2:**
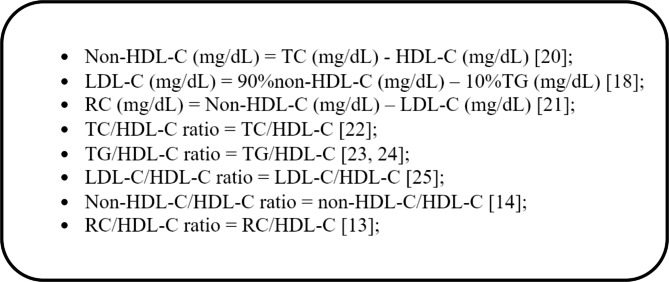
The calculation formulas of these lipid indicators. HDL-C: high-density lipoprotein cholesterol; non-HDL-C: non-high-density lipoprotein cholesterol; TC: total cholesterol; TG: triglyceride; LDL-C: low-density lipoprotein cholesterol; RC: remnant cholesterol

The calculation formulas of these lipid indicators were shown in Fig. 2 [13, 14, 18, 20–25].

### Determination of NAFLD

All subjects underwent abdominal ultrasonography. Gastroenterologists reviewed ultrasound images without knowledge of the biochemical examination and clinical information of the subjects, and made a comprehensive assessment and final diagnosis based on four ultrasound results including liver-kidney echo contrast, liver brightness, vascular blur, and depth attenuation [29].

### Statistical analysis

All analyses were done in R language version 3.4.3 and Empower(R) version 2.20. Baseline characteristics, according to the type of the variables and their distribution patterns, were summed up as the median (interquartile range), frequency (percentage), or mean (standard deviation). Mann-Whitney U test or t-test or chi-square test was used to check the differences between groups.

We first performed a univariate analysis of all variables to assess the correlation with NAFLD and then developed multivariate logistic regression models to calculate the odds ratio (ORs) of all lipid indicators to NAFLD and the corresponding 95% confidence intervals (CIs). To standardize the expression of OR values, we transformed 11 lipid indicators into Z-scores and using multiple linear regression checked the collinearity of covariates (Supplementary Table 1) [30]. Based on the Strengthening the Reporting of Observational Studies in Epidemiology (STROBE) statement, we ran three multivariate logistic regression models [31], in which, model 1 adjusted for the most important non-collinear demographic characteristics variables (adjusted sex, age, height, BMI, and WC), model 2 adjusted for blood pressure, drinking status, smoking status and exercise habits based on model 1, and model 3 made additional adjustments to the laboratory parameters (ALT, AST, GGT, FPG, and HbA1c) on the basis of model 2. Furthermore, to evaluate the ability and accuracy of 11 lipid indicators to identify NAFLD, we plotted the ROC curves for 11 lipid indicators and calculated the AUCs and optimal cut-off values.

## Results

### Prevalence and intergroup characteristics of NAFLD

After applying exclusion criteria, 14,251 adults were included in the analysis sample. Table 1 compares the baseline characteristics of the study population based on whether NAFLD was diagnosed or not, and the specific results were summarized as follows: (1) Except for drinking status, all baseline characteristics were significantly different between the two groups. (2) The prevalence of NAFLD was 17.59%, and among NAFLD patients, the proportion of males was much higher than that of females (80.93% vs. 19.07%), and they were also slightly older than the non-NAFLD subjects (44 vs. 42). (3) In terms of biomarkers, except HDL-C, all parameters including lipid indicators, liver enzyme metabolism indicators, and glucose metabolism indicators were significantly increased in the NAFLD group, with obvious adverse metabolic characteristics; among lipid indicators, non-traditional lipid indicators generally showed bigger differences between the two groups than traditional lipid indicators. (4) Participants with NAFLD were less likely to exercise and drink alcohol, while relatively more likely to smoke.

**Table 1:** Characteristics of the study subjects with and without NAFLD.

	Non-NAFLD	NAFLD	*P*-value
No of subjects	11,744	2507	
Sex			< 0.01
Women	6362 (54.17%)	478 (19.07%)	
Men	5382 (45.83%)	2029 (80.93%)	
Age, years	42.00 (18.00–79.00)	44.00 (19.00–72.00)	< 0.01
Weight, kg	57.72 (9.98)	72.18 (11.33)	< 0.01
Height, cm	164.11 (8.44)	168.03 (7.90)	< 0.01
BMI, kg/m^2^	21.33 (2.61)	25.50 (3.13)	< 0.01
WC, cm	74.09 (7.92)	85.98 (7.79)	< 0.01
ALT, IU/L	15.00 (2.00-856.00)	27.00 (6.00-220.00)	< 0.01
AST, IU/L	17.00 (3.00-590.00)	20.00 (6.00-140.00)	< 0.01
GGT, IU/L	14.00 (3.00-259.00)	23.00 (6.00-375.00)	< 0.01
TC, mmol/L	5.06 (0.85)	5.44 (0.87)	< 0.01
HDL-C, mmol/L	1.52 (0.40)	1.19 (0.29)	< 0.01
LDL-C. mmol/L	2.95 (0.95–9.72)	3.49 (1.16–6.59)	< 0.01
TG, mmol/L	0.65 (0.07–10.27)	1.24 (0.16–7.69)	< 0.01
Non-HDL-C, mmol/L	3.47 (1.22–10.93)	4.25 (1.38–7.71)	< 0.01
RC, mmol/L	0.50 (0.17–2.72)	0.71 (0.18–2.37)	< 0.01
TC/HDL-C ratio	3.33 (1.51–13.02)	4.71 (1.59–10.75)	< 0.01
TG/HDL-C ratio	0.43 (0.03–16.55)	1.07 (0.12–11.67)	< 0.01
LDL-C/HDL-C ratio	1.99 (0.43–10.35)	3.04 (0.50–8.01)	< 0.01
Non-HDL-C/HDL-C ratio	2.33 (0.51–12.02)	3.71 (0.59–9.75)	< 0.01
RC/HDL-C	0.33 (0.07–4.39)	0.62 (0.09–3.57)	< 0.01
FPG, mmol/L	5.09 (0.40)	5.39 (0.36)	< 0.01
HbA1c, %	5.15 (0.31)	5.30 (0.33)	< 0.01
SBP, mmHg	111.91 (14.02)	123.41 (14.83)	< 0.01
DBP, mmHg	69.69 (9.85)	77.81 (10.19)	< 0.01
Exercise habits	2093 (17.82%)	377 (15.04%)	< 0.01
Drinking status			0.16
no or rarely	9717 (82.74%)	2088 (83.29%)	
light	1472 (12.53%)	286 (11.41%)	
moderate	555 (4.73%)	133 (5.31%)	
Smoking status			< 0.01
Non	7561 (64.38%)	1185 (47.27%)	
Former	1920 (16.35%)	639 (25.49%)	
Current	2263 (19.27%)	683 (27.24%)	

Values were expressed as mean (SD) or medians (quartile interval) or n (%). Abbreviations: NAFLD: non-alcoholic fatty liver disease; BMI: body mass index; WC: waist circumference; ALT: alanine aminotransferase; AST: aspartate aminotransferase; GGT: gamma-glutamyl transferase; HDL-C: high-density lipoprotein cholesterol; TC: total cholesterol; TG: triglyceride; LDL-C: low density lipoprotein cholesterol; Non-HDL-C: non-high-density lipoprotein cholesterol; RC: remnant cholesterol; HbA1c: hemoglobin A1c; FPG: fasting plasma glucose; SBP: systolic blood pressure; DBP: diastolic blood pressure

### Association of TNNT lipid indicators with NAFLD

Table 2 shows the correlation between 11 lipid indicators and NAFLD and based on the STROBE statement we ran three multivariate logistic regression models; it can be seen that there were some slight changes in the degree of associations between TNNT lipid indicators and NAFLD in the three step-adjusted models, while the direction of the association remained consistent. In the final model, all lipid indicators were independently positively correlated with NAFLD, except HDL-C, which was negatively correlated with NAFLD. It is worth mentioning that RC/HDL-C ratio and RC were most strongly associated with NAFLD among all lipid indicators. Furthermore, by comparing the normalized OR values ​​corresponding to lipid indicators, we also found that the OR values ​​corresponding to traditional lipid indicators, except TG, were generally smaller than non-traditional lipid indicators.

**Table 2:** Association of NAFLD with the level of lipid-related parameters

	OR (95%CI)		
	Model I	Model II	Model II
TC	1.35 (1.27, 1.43)	1.32 (1.24, 1.41)	1.15 (1.07, 1.23)
HDL-C	0.29 (0.24, 0.35)	0.29 (0.24, 0.35)	0.33 (0.27, 0.40)
LDL-C	1.51 (1.40, 1.62)	1.46 (1.35, 1.57)	1.24 (1.14, 1.34)
TG	2.14 (1.97, 2.32)	2.15 (1.98, 2.34)	1.88 (1.72, 2.05)
Non-HDL-C	1.57 (1.47, 1.67)	1.53 (1.44, 1.64)	1.32 (1.23, 1.41)
RC	14.54 (11.09, 19.06)	14.27 (10.84, 18.78)	8.19 (6.12, 10.94)
TC/HDL-C ratio	1.50 (1.43, 1.58)	1.50 (1.43, 1.57)	1.36 (1.29, 1.43)
TG/HDL-C ratio	1.83 (1.70, 1.97)	1.84 (1.71, 1.98)	1.64 (1.52, 1.77)
LDL-C/HDL-C ratio	1.58 (1.49, 1.68)	1.57 (1.48, 1.67)	1.39 (1.31, 1.49)
Non-HDL-C/HDL-C ratio	1.50 (1.43, 1.58)	1.50 (1.43, 1.57)	1.36 (1.29, 1.43)
RC/HDL-C ratio	6.61 (5.38, 8.12)	6.68 (5.41, 8.25)	4.53 (3.63, 5.64)

Abbreviations: OR: Odds ratios; other abbreviations as in Table ​1

Model I adjusted sex, age, height, BMI and WC.

Model II adjusted model I + SBP, DBP, exercise habits, smoking status and Drinking status

Model III adjusted model II + ALT, AST, GGT, FPG and HbA1c.

### Accuracy of TNNT lipid indicators in identifying NAFLD in the general population

**Fig. 3:**
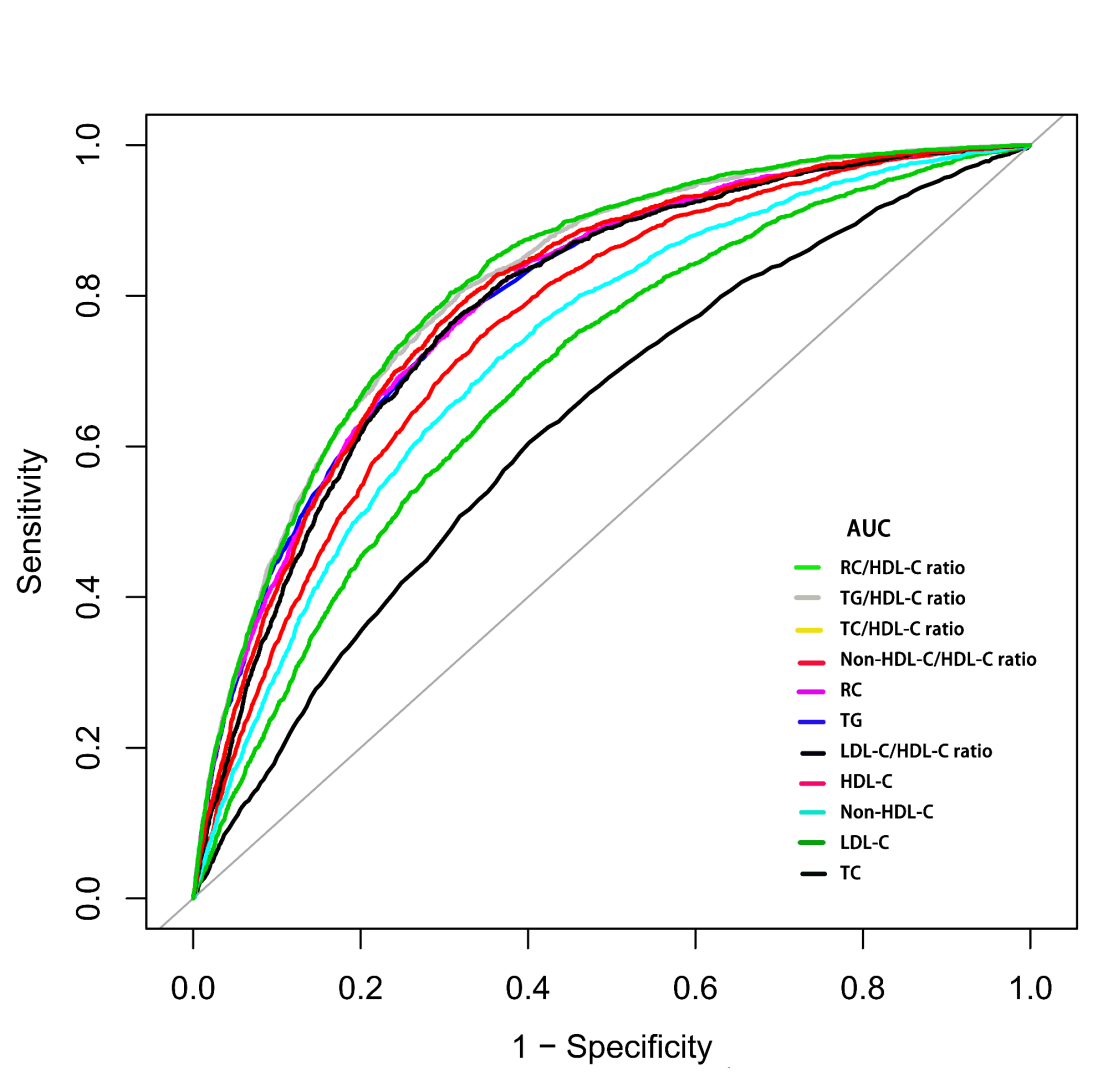
Receiver operating characteristic curve analysis of NAFLD-related lipid indicators. NAFLD: nonalcoholic fatty liver disease; AUC: area under the curve; HDL-C: high-density lipoprotein cholesterol; non-HDL-C: non-high-density lipoprotein cholesterol; TC: total cholesterol; TG: triglyceride; LDL-C: Low-density lipoprotein cholesterol; RC: remnant cholesterol

To compare the accuracy of TNNT lipid indicators to identify NAFLD, we plotted the ROC curves of 11 lipid indicators (Fig. 3) and calculated the AUCs, optimal cut-off values, specificity, and sensitivity (Table 3). The results showed that all 11 lipid indicators had AUCs greater than 0.5, among which the non-traditional lipid indicators RC/HDL-C ratio and TG/HDL-C ratio had the best ability to identify NAFLD, while LDL-C and TC performed poorly in traditional lipid indicators. Moreover, we also calculated the optimal cut-off value of the TG/HDL-C ratio for identifying NAFLD was 0.61 and that of the RC/HDL-C ratio was 0.43.

**Table 3:** The best threshold, sensitivities, specificities, and area under the curve of lipid-related parameters for the screening of NAFLD in the general population

	AUC	95%CI low	95%CI upp	Best threshold	Specificity	Sensitivity
TC	0.63	0.62	0.64	5.21	0.60	0.61
HDL-C	0.76	0.75	0.77	1.34	0.65	0.75
LDL-C	0.69	0.68	0.71	3.05	0.56	0.74
TG	0.80	0.79	0.81	0.84	0.68	0.77
Non-HDL-C	0.73	0.72	0.74	3.77	0.64	0.72
RC	0.80	0.79	0.81	0.58	0.69	0.76
TC/HDL-C ratio	0.80	0.79	0.81	3.80	0.67	0.80
TG/HDL-C ratio	0.81	0.81	0.82	0.61	0.68	0.80
LDL-C/HDL-C ratio	0.79	0.78	0.80	2.43	0.69	0.76
Non-HDL-C/HDL-C ratio	0.80	0.79	0.81	2.80	0.67	0.80
RC/HDL-C ratio	0.82	0.81	0.83	0.43	0.69	0.80

Abbreviations: AUC: area under the curve; other abbreviations as in Table ​1

**Assessing the accuracy of all lipid indicators for identifying NAFLD across sex, age, and BMI categories**.

We also explored the ability of all lipid indicators to identify NAFLD in different populations, stratified by sex, age, and BMI, respectively (Tables 4, 5 and 6). First, we analyzed the accuracy of 11 lipid indicators for assessing NAFLD in different sexes (Table 4). In the female group, the AUC values ​​of TG, RC, TG/HDL-C ratio and RC/HDL-C ratio were larger, all exceeding 0.80, and their optimal cut-off values ​​were 0.72, 0.54, 0.50, and 0.38, respectively. In the male group, the AUCs ​​of lipid indicators such as LDL-C/HDL-C ratio, RC, RC/HDL-C ratio, non-HDL-C/HDL-C ratio, TC/HDL-C ratio, TG and TG/HDL-C ratio were larger, all of them larger than 0.70, of which the RC/HDL-C ratio was the best lipid indicator for identifying NAFLD (AUC = 0.75), and its optimal cut-off value was 0.51. In contrast, TC had a modest ability to recognize NAFLD in both male and female groups.

**Table 4:** The best threshold, sensitivities, specificities, and area under the curve of lipid-related parameters for the screening of NAFLD in man and women

	AUC	95%CI low	95%CI upp	Best threshold	Specificity	Sensitivity
Women						
TC	0.66	0.64	0.69	5.24	0.61	0.65
HDL-C	0.72	0.70	0.75	1.46	0.69	0.67
LDL-C	0.72	0.70	0.74	3.05	0.62	0.73
TG	0.80	0.79	0.82	0.72	0.70	0.77
Non-HDL-C	0.75	0.72	0.77	3.55	0.62	0.77
RC	0.81	0.79	0.83	0.54	0.72	0.77
TC/HDL-C ratio	0.79	0.77	0.81	3.41	0.70	0.77
TG/HDL-C ratio	0.82	0.80	0.84	0.50	0.75	0.75
LDL-C/HDL-C ratio	0.78	0.76	0.80	2.24	0.77	0.70
Non-HDL-C/HDL-C ratio	0.79	0.77	0.81	2.41	0.70	0.77
RC/HDL-C ratio	0.82	0.80	0.84	0.38	0.77	0.75
Men						
TC	0.62	0.61	0.63	5.21	0.59	0.59
HDL-C	0.69	0.67	0.70	1.27	0.54	0.75
LDL-C	0.65	0.64	0.67	3.39	0.67	0.56
TG	0.74	0.72	0.75	1.07	0.70	0.65
Non-HDL-C	0.69	0.67	0.70	3.85	0.60	0.70
RC	0.74	0.73	0.75	0.63	0.67	0.70
TC/HDL-C ratio	0.73	0.72	0.75	4.21	0.64	0.72
TG/HDL-C ratio	0.75	0.74	0.76	0.84	0.67	0.70
LDL-C/HDL-C ratio	0.72	0.71	0.73	2.75	0.66	0.68
Non-HDL-C/HDL-C ratio	0.73	0.72	0.75	3.21	0.64	0.72
RC/HDL-C ratio	0.75	0.74	0.77	0.51	0.65	0.73

Abbreviations: AUC: area under the curve; other abbreviations as in Table ​1

Table 5 shows the ability of 11 lipid indicators to identify NAFLD after stratification by age. In the younger age group (< 30 years old), lipid indicators that performed very well (AUCs greater than 0.90) for the identification of NAFLD were the LDL-C/HDL-C ratio, TG, RC, non-HDL-C/HDL-C ratio, TC/HDL-C ratio, RC/HDL-C ratio, TG/HDL-C ratio; additionally, LDL-C, HDL-C and non-HDL-C also performed well in identifying NAFLD (AUCs were all greater than 0.8). In the middle-aged population (30–44 years old), the LDL-C/HDL-C ratio, TG, RC, non-HDL-C/HDL-C ratio, TC/HDL-C ratio, TG/HDL-C ratio, RC/HDL-C ratio were good lipid indicators for identifying NAFLD, and their AUC values ​​all exceed 0.80, with RC/HDL-C ratio being the best lipid indicator (AUC = 0.85). And in the 45–59 age group, TG/HDL-C ratio was the best lipid indicator used to identify NAFLD (AUC: 0.78). In contrast, in the elderly population (≥ 60 years old), the AUC values of all 11 lipid indicators were lower than those of other age subgroups, and the AUC values of all lipid indicators ranged from 0.50 to 0.70, and the best lipid indicator for this age group was the RC/HDL-C ratio (AUC = 0.68).

**Table 5:** The best threshold, sensitivities, specificities, and area under the curve of lipid-related parameters for the screening of NAFLD in different age groups

	AUC	95%CI low	95%CI upp	Best threshold	Specificity	Sensitivity
< 30 years old						
TC	0.73	0.60	0.86	4.90	0.76	0.68
HDL-C	0.85	0.76	0.94	1.09	0.92	0.68
LDL-C	0.82	0.72	0.92	2.85	0.75	0.86
TG	0.91	0.87	0.96	0.75	0.84	0.86
Non-HDL-C	0.85	0.77	0.93	3.63	0.85	0.77
RC	0.92	0.87	0.97	0.53	0.84	0.91
TC/HDL-C ratio	0.92	0.87	0.97	3.86	0.89	0.82
TG/HDL-C ratio	0.93	0.89	0.97	0.44	0.73	1.00
LDL-C/HDL-C ratio	0.91	0.86	0.96	2.09	0.76	0.91
Non-HDL-C/HDL-C ratio	0.92	0.87	0.97	2.86	0.89	0.82
RC/HDL-C ratio	0.93	0.89	0.98	0.40	0.85	0.86
30–44 years old						
TC	0.67	0.65	0.69	5.21	0.70	0.57
HDL-C	0.78	0.77	0.80	1.34	0.66	0.79
LDL-C	0.73	0.72	0.75	3.12	0.68	0.67
TG	0.83	0.81	0.84	0.85	0.74	0.76
Non-HDL-C	0.77	0.76	0.78	3.77	0.73	0.69
RC	0.83	0.82	0.84	0.55	0.70	0.82
TC/HDL-C ratio	0.83	0.82	0.84	3.70	0.70	0.83
TG/HDL-C	0.84	0.83	0.85	0.63	0.74	0.80
LDL-C/HDL-C ratio	0.82	0.81	0.83	2.27	0.69	0.82
Non-HDL-C/HDL-C ratio	0.83	0.82	0.84	2.70	0.70	0.83
RC/HDL-C ratio	0.85	0.84	0.86	0.40	0.70	0.85
45–59 years old						
TC	0.56	0.54	0.58	5.21	0.46	0.64
HDL-C	0.74	0.72	0.75	1.30	0.68	0.69
LDL-C	0.63	0.61	0.65	3.46	0.63	0.57
TG	0.76	0.74	0.77	0.94	0.67	0.71
Non-HDL-C	0.67	0.65	0.68	4.04	0.62	0.64
RC	0.75	0.73	0.76	0.63	0.69	0.70
TC/HDL-C ratio	0.75	0.74	0.77	4.11	0.70	0.71
TG/HDL-C ratio	0.78	0.76	0.79	0.73	0.70	0.73
LDL-C/HDL-C ratio	0.74	0.73	0.76	2.47	0.63	0.75
Non-HDL-C/HDL-C ratio	0.75	0.74	0.77	3.11	0.70	0.71
RC/HDL-C ratio	0.78	0.76	0.79	0.43	0.62	0.80
≥ 60 years old						
TC	0.56	0.50	0.62	5.29	0.43	0.72
HDL-C	0.64	0.58	0.69	1.34	0.56	0.69
LDL-C	0.61	0.55	0.66	3.13	0.40	0.79
TG	0.67	0.61	0.73	0.98	0.62	0.64
Non-HDL-C	0.64	0.58	0.69	4.42	0.72	0.50
RC	0.67	0.62	0.73	0.63	0.61	0.65
TC/HDL-C ratio	0.67	0.62	0.72	3.84	0.52	0.76
TG/HDL-C ratio	0.68	0.62	0.73	0.91	0.75	0.53
LDL-C/HDL-C ratio	0.66	0.61	0.72	2.46	0.54	0.75
Non-HDL-C/HDL-C ratio	0.67	0.62	0.72	2.84	0.52	0.76
RC/HDL-C ratio	0.68	0.63	0.74	0.48	0.64	0.64

Abbreviations: AUC: area under the curve; other abbreviations as in Table ​1

Finally, we also conducted a stratified analysis based on BMI (Table 6). In the low-weight population, RC and TG had the same ability to identify NAFLD, with AUCs greater than 0.90, and their optimal cut-off values ​​were 0.59 and 0.88, respectively. Besides, LDL-C, non-HDL-C, TG/HDL-C ratio and TC also had a good ability to identify NAFLD, and their AUC values ​​were all greater than 0.85. In the normal-weight population, the RC/HDL-C ratio, with an AUC of 0.78, was the optimal lipid indicator for the identification of NAFLD. In the overweight/obese population, the ability of 11 lipid indicators to identify NAFLD was mediocre (all AUCs less than 0.70).

**Table 6:** The best threshold, sensitivities, specificities, and area under the curve of lipid-related parameters for the screening of NAFLD in different BMI groups

	AUC	95%CI low	95%CI upp	Best threshold	Specificity	Sensitivity
Low weight (BMI < 18.5 kg/m^2^)						
TC	0.85	0.66	1.00	5.11	0.65	1.00
HDL-C	0.63	0.12	1.00	2.18	0.87	0.67
LDL-C	0.86	0.83	0.89	3.27	0.84	1.00
TG	0.93	0.89	0.97	0.88	0.89	1.00
Non-HDL-C	0.88	0.84	0.92	3.85	0.85	1.00
RC	0.93	0.89	0.97	0.59	0.90	1.00
TC/HDL-C ratio	0.66	0.39	0.93	2.81	0.52	1.00
TG/HDL-C ratio	0.86	0.79	0.93	0.48	0.81	1.00
LDL-C/HDL-C ratio	0.64	0.35	0.93	1.51	0.48	1.00
Non-HDL-C/HDL-C ratio	0.66	0.39	0.93	1.81	0.52	1.00
RC/HDL-C ratio	0.78	0.61	0.94	0.29	0.68	1.00
Normal weight (BMI:18.5–24.9 kg/m^2^)						
TC	0.62	0.60	0.63	5.16	0.57	0.62
HDL-C	0.72	0.71	0.73	1.40	0.57	0.76
LDL-C	0.67	0.66	0.69	3.04	0.54	0.72
TG	0.77	0.76	0.78	0.84	0.68	0.73
Non-HDL-C	0.70	0.69	0.72	3.77	0.63	0.68
RC	0.77	0.75	0.78	0.56	0.63	0.78
TC/HDL-C ratio	0.76	0.75	0.78	3.80	0.67	0.75
TG/HDL-C ratio	0.78	0.77	0.80	0.60	0.67	0.76
LDL-C/HDL-C ratio	0.75	0.74	0.77	2.30	0.64	0.77
Non-HDL-C/HDL-C ratio	0.76	0.75	0.78	2.80	0.67	0.75
RC/HDL-C ratio	0.78	0.77	0.80	0.40	0.64	0.80
Overweight/Obese (BMI ≥ 25 kg/m^2^)						
TC	0.57	0.55	0.60	5.52	0.65	0.47
HDL-C	0.64	0.61	0.66	1.22	0.54	0.69
LDL-C	0.59	0.56	0.61	3.62	0.68	0.47
TG	0.68	0.66	0.70	1.24	0.71	0.56
Non-HDL-C	0.62	0.60	0.64	4.00	0.53	0.66
RC	0.68	0.66	0.70	0.73	0.73	0.53
TC/HDL-C ratio	0.67	0.64	0.69	4.59	0.64	0.62
TG/HDL-C ratio	0.69	0.67	0.71	0.96	0.64	0.65
LDL-C/HDL-C ratio	0.65	0.63	0.68	2.75	0.55	0.70
Non-HDL-C/HDL-C ratio	0.67	0.64	0.69	3.59	0.64	0.62
RC/HDL-C ratio	0.69	0.67	0.71	0.57	0.61	0.67

Abbreviations: AUC: area under the curve; other abbreviations as in Table ​1

## Discussion

The current study comprehensively assessed the associations and diagnostic values of 11 TNNT lipid indicators with NAFLD. The main findings were summarized as follows: (1) TNNT lipid indicators were independently associated with NAFLD, with RC and the RC/HDL-C ratio having the strongest association with NAFLD. (2) Non-traditional lipid indicators were used more accurately than traditional lipid indicators to identify NAFLD, with RC/HDL-C ratio and TG/HDL-C ratio having the highest AUCs. (3) Compared with men, non-traditional lipid indicators had higher diagnostic values for NAFLD in women. (4) In the younger age group (< 30 years old), all non-traditional lipid indicators presented an excellent (AUCs > 0.90) ability to identify NAFLD, except for non-HDL (AUC = 0.85). (5) All lipid indicators had a higher recognition ability for NAFLD in low-weight populations compared to normal-weight or overweight/obese populations.

With economic development, and changes in lifestyle and dietary habits [32], NAFLD has gradually become an increasingly huge and serious global public health problem and has brought a huge social and economic burden, seriously endangering human health [3–8, 33–36]. The pathogenesis of NAFLD is complex and atherogenic dyslipidemia is now known to be an extremely important risk factor for NAFLD, with elevated atherogenic lipid indicators LDL-C, TG, and decreased HDL-C commonly observed in NAFLD patients [9–12]. The mechanisms by which lipid abnormalities lead to NAFLD are numerous and complex, with insulin resistance (IR) playing a huge role [37, 38]. Generally speaking, in the state of hyperinsulinemia and hyperglycemia, the release of free fatty acids from adipocytes increases, and the de novo synthesis of liver fat and the concentrations of TG in serum increase, which further promotes the hepatic synthesis and secretion of very-low-density lipoprotein in large amounts and accelerates the hepatic fat accumulation [39, 40]; in addition, reduced hydrolysis of TG and weakened synthesis of HDL-C in response to IR result in increased serum TG concentrations as well as reduced HDL-C concentrations [39, 41, 42]. These atherogenic lipid abnormalities present an elevated risk of developing NAFLD; therefore, monitoring and screening for atherogenic lipid indicators are beneficial for the primary prevention of NAFLD.

### Comparisons with other studies and what does the current work add to the existing knowledge

The traditional lipid indicators HDL-C, LDL-C, TG and TC have been shown to be directly or indirectly associated with NAFLD in previous studies [16–19], and several intervention studies have indicated that active lipid management in patients with NAFLD was effective in reducing the relative risk of cardiovascular events [43, 44]. In the current study, the researchers found that TG may be the most valuable traditional lipid indicator for NAFLD screening. These findings further validated the previous findings and provided a reliable reference for the use of TG as a traditional lipid indicator in NAFLD screening and treatment.

Non-traditional lipid indicators have been a research hotspot in recent years, and numerous studies have demonstrated a strong association between non-traditional lipid indicators and NAFLD [13, 14, 22–25], and in general, non-traditional lipid indicators have good application in the risk assessment of NAFLD. However, it is not clear which non-traditional lipid indicators are the most valuable ones for identifying NAFLD. According to a recent study by Zou et al., RC may be a better lipid parameter than non-HDL-C and traditional lipid indicators in identifying NAFLD [21], but only two non-traditional lipid indicators were considered in their study, and the diagnostic values of other non-traditional lipid indicators in NAFLD need to be further evaluated and compared. As a continuation and deepening of the study by Zou et al., in the present study, the researchers analyzed the diagnostic values of 7 non-traditional lipid indicators for NAFLD and showed that RC/HDL-C ratio was the best non-traditional lipid indicator for identifying NAFLD, especially in young and middle-aged adults and women. It should also be noted that with the exception of the RC/HDL-C ratio, TG/HDL-C ratio was the optimal non-traditional lipid indicator for identifying NAFLD. Several pieces of evidence have previously demonstrated that the TG/HDL-C ratio was a useful IR surrogate with significant advantages in the identification of metabolic diseases, including NAFLD [23, 24]. However, in a recent cross-sectional study of patients who underwent bariatric surgery, a different result was reported. In the study by Cazzo et al. who analyzed 89 patients who underwent bariatric surgery, they found that the risk of NAFLD in morbidly obese individuals was not associated with the TG/HDL-C ratio [45]. In the current study, the researchers found that the TG/HDL-C ratio was not only independently associated with NAFLD but also had a certain diagnostic value in overweight/obese people. The discrepancy between the results of the current study and those of Cazzo et al. may be related to the fact that their population underwent bariatric surgery, which resulted in some degree of improvement in the lipid profile of the patients and a significant reduction in hepatic steatosis.

In this study, the researchers also performed a stratified ROC analysis by sex, age, and BMI and found some interesting results: (1) Compared with traditional lipid indicators, most of the non-traditional lipid indicators were better at identifying female NAFLD, which may be related to the difference in body fat distribution between the sexes. It has been reported [46, 47] that during evolution, women tend to store fat in subcutaneous adipose tissue in order to cope with the risk of nutritional deficiencies during lactation, while men tend to store fat more in visceral adipose tissue, and the accumulation of visceral fat would reduce serum adiponectin, which may increase the risk of NAFLD. (2) Most of the non-traditional lipid indicators were more suitable for identifying NAFLD in young people, which may be related to more and more unhealthy eating habits and living habits of young people. A longitudinal cohort study with a median follow-up of 23 years reported that childhood obesity significantly increased the risk of NAFLD in adulthood [48]. Not only that, some unhealthy lifestyles, such as sedentary, skipping breakfast, and lack of sleep are all problems faced by contemporary young people, which also greatly contribute to the prevalence of obesity and NAFLD [49, 50]. Furthermore, studies on genetics and nutrition had shown that infants who had breastfed for less than 6 months, mothers who were obese in early pregnancy, or infants who were obese in adolescence had a significantly increased risk of NAFLD at age 17 [51], which also indicated that the risk of NAFLD began from infancy and early childhood. (3) All lipid indicators were of greater value in low-weight groups. Several recent studies have shown that non-obese groups were more prone to metabolic disorders, and not only that, dysbiosis of the gut flora and genetic susceptibility were also involved [52, 53]. In addition, it is also important to note that approximately 2/5 of NAFLD population worldwide is classified as non-obese [54]. Therefore, clinical practice should provide early public health education to these special populations and develop effective interventions to reduce the prevalence of NAFLD and the corresponding complications.

### Study strengths and limitations

This study is the first to comprehensively compare the degree of associations of 11 TNNT lipid indicators with NAFLD and the accuracy of identifying NAFLD and found that the value of non-traditional lipid indicators for identifying NAFLD was superior to traditional lipid indicators, especially the RC/HDL-C ratio and the TG/HDL-C ratio. Secondly, the study calculated optimal cut-off values and AUCs for lipid indicators used to identify NAFLD in different populations, stratified by sex, age, and BMI, and these exploratory stratification analyses provided new insights for precision medicine efforts.

This study also has some limitations that must be mentioned. (1) Considering that the current study used a cross-sectional design, thus causal associations could not be explained. (2) The subjects of this study were Japanese adults, so the external applicability of the study results needs to be confirmed by further studies, and therefore the results of this study are only for reference to other ethnic groups. (3) In this study, abdominal ultrasound was used to diagnose NAFLD, which may have been missed in some patients compared with liver biopsy. (4) The potential relationship of the RC/HDL-C ratio with NAFLD may be mediated by IR, however, data related to the measurement of IR were lacking in the current study and further studies are needed.

## Conclusion

In conclusion, in the Japanese population, non-traditional lipid indicators had a higher diagnostic value for NAFLD compared to traditional lipid indicators, and lipid indicators alone had a lower diagnostic value for NAFLD than the ratio of two lipid indicators, with the RC/HDL-C ratio and TG/HDL-C ratio being the best lipid indicators for identifying NAFLD. Considering that the calculation of non-traditional lipid indicators is simple, convenient, and easy to promote, it is recommended that non-traditional lipid indicators be used more often as routine monitoring indicators as well as non-invasive assessment methods in future clinical practice.

## Electronic supplementary material

Below is the link to the electronic supplementary material.

Supplementary Material 1

## Data Availability

The data used in this study have been uploaded to the “Dryad” database (10.5061/dryad.1n6c4) by Professor Okamura et al.

## Abbreviations

(NAFLD): Nonalcoholic fatty liver disease
(TNNT): Traditional and non-traditional
(TC): Total cholesterol
(HDL-C): High-density lipoprotein cholesterol
(LDL-C): Low-density lipoprotein cholesterol
(TG): Triglyceride
(RC): Remnant cholesterol
(ROC): Eceiver operating characteristic
(AUCs): Area under the curves
(NAGALA): NAfld in Gifu Region, Japan, Lorngitudinal Analysis
(WC): Waist circumference
(BMI): Body mass index
(GGT): γ-glutamyl transferase
(AST): Aspartate aminotransferase
(HbA1c): Glycosylated hemoglobin A1c
(ALT): Alanine aminotransferase
(FPG): Fasting plasma glucose
(OR): Odds ratio
(CI): Confidence intervals
(IR): Insulin resistance
