# Supplementary material for: Utility of traditional and non-traditional lipid indicators in the diagnosis of nonalcoholic fatty liver disease in a Japanese population
Source: Lipids Health Dis. 2022 Oct 7;21:95. doi: 10.1186/s12944-022-01712-z (PMC9540727; doi:10.1186/s12944-022-01712-z)
Supplement: Supplementary file 1 — Supplementary Material 1 [file 12944_2022_1712_MOESM1_ESM.docx]

Supplementary Table 1: Collinearity diagnostics steps.

|  | VIF | | | | | | | | | |
| --- | --- | --- | --- | --- | --- | --- | --- | --- | --- | --- |
|  | Step 1 | Step 2 | Step 3 | Step 4 | Step 5 | Step 6 | Step 7 | Step 8 | Step 9 | Step 10 |
| Gender | 3.3 | 3.3 | 3.3 | 3.3 | 3.3 | 3.3 | 3.3 | 3.3 | 3.3 | 3.3 |
| Age | 1.4 | 1.4 | 1.4 | 1.4 | 1.4 | 1.4 | 1.4 | 1.4 | 1.4 | 1.4 |
| Weight | 169.4 | 169.4 | 169.4 | 169.4 | 169.4 | 169.4 | 169.4 | NA | NA | NA |
| Height | 52.2 | 52.2 | 52.2 | 52.2 | 52.2 | 52.2 | 52.2 | 2.9 | 2.9 | 2.9 |
| BMI | 96.4 | 96.4 | 96.4 | 96.4 | 96.4 | 96.4 | 96.4 | 5 | 5 | 5 |
| WC | 5.9 | 5.9 | 5.9 | 5.9 | 5.9 | 5.9 | 5.9 | 5.9 | 5.9 | 5.9 |
| ALT | 4.2 | 4.2 | 4.2 | 4.2 | 4.2 | 4.2 | 4.2 | 4.1 | 4.1 | 4.1 |
| AST | 3.3 | 3.3 | 3.3 | 3.3 | 3.3 | 3.3 | 3.3 | 3.3 | 3.3 | 3.3 |
| GGT | 1.5 | 1.5 | 1.5 | 1.5 | 1.5 | 1.5 | 1.5 | 1.5 | 1.5 | 1.5 |
| TC | Inf | NA | NA | NA | NA | NA | NA | NA | NA | NA |
| HDL.C | Inf | 5.4 | 5.4 | 5.4 | 5.4 | 5.4 | 5.4 | 5.4 | 4.8 | 1.6 |
| LDL.C | Inf | Inf | NA | NA | NA | NA | NA | NA | NA | NA |
| TG | Inf | Inf | Inf | NA | NA | NA | NA | NA | NA | NA |
| NON-HDL-C | Inf | Inf | Inf | 26.9 | 26.9 | 26.9 | 26.9 | 26.9 | 5.3 | 3 |
| RC | Inf | Inf | Inf | 39.5 | 39.5 | 39.5 | 39.5 | 39.5 | 4 | 3.5 |
| TC/HDL-C ratio | Inf | Inf | Inf | Inf | NA | NA | NA | NA | NA | NA |
| TG/HDL-C ratio | Inf | Inf | Inf | Inf | Inf | NA | NA | NA | NA | NA |
| LDL-C/HDL-C ratio | Inf | Inf | Inf | Inf | Inf | Inf | NA | NA | NA | NA |
| NON-HDL-C/HDL-C ratio | Inf | Inf | Inf | Inf | Inf | Inf | 57.7 | 57.6 | 11.9 | NA |
| RC/HDL-C ratio | Inf | Inf | Inf | Inf | Inf | Inf | 64.7 | 64.7 | NA | NA |
| FPG | 1.5 | 1.5 | 1.5 | 1.5 | 1.5 | 1.5 | 1.5 | 1.5 | 1.5 | 1.5 |
| HbA1c | 1.2 | 1.2 | 1.2 | 1.2 | 1.2 | 1.2 | 1.2 | 1.2 | 1.2 | 1.2 |
| SBP | 5.5 | 5.5 | 5.5 | 5.5 | 5.5 | 5.5 | 5.5 | 5.5 | 5.5 | 5.5 |
| DBP | 5.6 | 5.6 | 5.6 | 5.6 | 5.6 | 5.6 | 5.6 | 5.6 | 5.6 | 5.6 |
| Exercise habits | 1 | 1 | 1 | 1 | 1 | 1 | 1 | 1 | 1 | 1 |
| Drinking status | 1.2 | 1.2 | 1.2 | 1.2 | 1.2 | 1.2 | 1.2 | 1.2 | 1.2 | 1.2 |
| Smoking status | 1.4 | 1.4 | 1.4 | 1.4 | 1.4 | 1.4 | 1.4 | 1.4 | 1.4 | 1.4 |

Abbreviations: Inf: infinity; VIF: Variance inflation factor; Other abbreviations as in Table ​1.

Note: VIF = 1/(1-R^2^).
